# Dietary Patterns of Breastfeeding Mothers and Human Milk Composition: Data from the Italian MEDIDIET Study

**DOI:** 10.3390/nu13051722

**Published:** 2021-05-19

**Authors:** Francesca Bravi, Matteo Di Maso, Simone R. B. M. Eussen, Carlo Agostoni, Guglielmo Salvatori, Claudio Profeti, Paola Tonetto, Pasqua Anna Quitadamo, Iwona Kazmierska, Elisabetta Vacca, Adriano Decarli, Bernd Stahl, Enrico Bertino, Guido E. Moro, Monica Ferraroni, on behalf of the MEDIDIET Working Group

**Affiliations:** 1 Department of Clinical Sciences and Community Health, Branch of Medical Statistics, Biometry and Epidemiology “G.A. Maccacaro”, Università degli Studi di Milano, 20133 Milan, Italy; matteo.dimaso@unimi.it (M.D.M.); adriano.decarli@unimi.it (A.D.); monica.ferraroni@unimi.it (M.F.); 2 Danone Nutricia Research, 3584 Utrecht, The Netherlands; simone.eussen@danone.com (S.R.B.M.E.); elisabetta.vacca@danone.com (E.V.); bernd.stahl@danone.com (B.S.); 3 Pediatric Intermediate Care Unit, Fondazione IRCCS Ospedale Cà Granda-Ospedale Maggiore Policlinico, 20122 Milan, Italy; carlo.agostoni@unimi.it; 4 Department of Clinical Science and Community Health, Università degli Studi di Milano, 20122 Milan, Italy; 5 Neonatal Intensive Care Unit, IRCCS Ospedale Pediatrico Bambino Gesù, 00165 Rome, Italy; salvatori.guglielmo@tiscali.it; 6 Neonatal Intensive Care Unit, Azienda Ospedaliera Universitaria Meyer di Firenze, 50139 Florence, Italy; claudio.profeti@meyer.it; 7 Neonatal Care Unit of the University, City of Health and Science Hospital, 10126 Turin, Italy; paola.tonetto@unito.it (P.T.); enrico.bertino@unito.it (E.B.); 8 Neonatology–Neonatal Intensive Care Unit, IRCCS Casa Sollievo della Sofferenza, 71013 San Giovanni Rotondo, Italy; pasquaq@tiscali.it; 9 Neonatal Intensive Care Unit, Ospedale Buccheri La Ferla Fatebenefratelli, 90123 Palermo, Italy; iwonalidiaka@gmail.com; 10 Department of Chemical Biology & Drug Discovery, Utrecht Institute for Pharmaceutical Sciences, Faculty of Science, Utrecht University, 3584 Utrecht, The Netherlands; 11 Italian Association of Human Milk Banks (AIBLUD), 20126 Milan, Italy; guidoemoro@tiscali.it

**Keywords:** maternal nutrition breastfeeding, dietary patterns, factor analysis, human milk

## Abstract

(1) Background: Several studies have reported associations between maternal diet in terms of single foods or nutrients and human milk compounds, while the overall role of maternal diet and related dietary patterns has rarely been investigated. (2) Methods: Between 2012 and 2014, we enrolled 300 healthy Italian mothers, who exclusively breastfed their infant. During a hospital visit at 6 weeks postpartum, a sample of freshly expressed foremilk was collected and information on maternal dietary habits in the postpartum period was obtained through an interviewer-administered food frequency questionnaire. We applied principal component factor analysis to selected nutrients in order to identify maternal dietary patterns, and assessed correlations in human milk macronutrients and fatty acids across levels of dietary patterns. (3) Results: Five dietary patterns were identified, named “Vitamins, minerals and fibre”, “Proteins and fatty acids with legs”, “Fatty acids with fins”, “Fatty acids with leaves”, “Starch and vegetable proteins”. These dietary patterns were correlated with some milk components, namely fatty acids, and in particular ω-3 and its subcomponents. (4) Conclusions: This study showed that overall maternal dietary habits during breastfeeding may influence human milk composition, suggesting the importance of adequate maternal nutrition during lactation not only for the mother herself but also to provide the infant with milk containing adequate amount and quality of nutrients for a balanced nutrition.

## 1. Introduction

Human milk provided by healthy and well-nourished mothers is the optimal way of providing nutrients as required by biological processes associated with growth and development [1]. It contains several bioactive compounds (e.g., nutrients, immune and stem cells, hormones, digestive enzymes, and macrophages) that contribute to the development of the gastrointestinal tract, immune system and brain of the infant [2,3,4]. Breastfeeding has several health benefits, both in the short-term (i.e., reducing neonatal and child mortality from infectious diseases) and in the long-term (i.e., preventing adult obesity, diabetes, cardiovascular diseases, and metabolic disorders) for the infant [5,6,7,8]. Moreover, the favorable role of the bioactive molecules in human milk may be of particular importance in preterm infants, who have higher morbimortality rates [9]. The World Health Organization encourages mothers to exclusively breastfeed their infants during the first six months of life, to achieve optimal growth, development and health parameters, and thereafter breastfeeding should be continued in conjunction with nutritionally adequate and safe complementary food until the child is up to two years old or beyond [1].

The composition of human milk depends on different environmental and maternal factors [10,11,12,13]. Maternal dietary habits have been reported to influence human milk fatty acids (FA) composition, while macronutrient contents in milk appear less sensitive to maternal dietary intakes [14,15]. Nevertheless, a systematic review reported that most of the included studies evaluated the relationship between maternal diet and FA profile in human milk and only few studies investigated the relationship with other macro- and micronutrient milk compounds [16]. Most studies have analysed the association of individual nutrients or foods with the levels of human milk compounds. However, dietary habits are based on a variety of foods, which in turn include complex combinations of nutrients. Dietary pattern analysis is a way to reduce the complexity of nutritional intake identifying a smaller number of factors, which are called dietary patterns. Dietary patterns analysis was widely used to assess the impact of diet on several health outcomes [17,18,19] and only recently was introduced to identify either patterns in maternal nutrition [15,20] or patterns in human milk composition [21,22,23,24].

Using data of the Italian MEDIDIET study, we evaluated the association between maternal dietary patterns and human milk macronutrient and fatty acid composition.

## 2. Materials and Methods

### 2.1. Study Design and Participants

The MEDIDIET study is an Italian multicentric observational study carried out between 2012 and 2014, including 300 healthy breastfeeding mothers. Study design, inclusion criteria, dietary assessment of breastfeeding mothers, milk collection, analysis of milk samples and description of study characteristics have been provided in detail elsewhere [25]. Briefly, mothers were enrolled in five participating hospitals as follows: 110 mothers from Turin, 23 from Florence, 46 from Rome, 101 from San Giovanni Rotondo, and 20 from Palermo. Mothers were aged 25–41 years old and gave birth to healthy term infants, who were exclusively breastfed from birth to the day of milk collection (i.e., 6 ± 1 week postpartum). To be enrolled in the study, mothers had to be healthy (i.e., free of diabetes, autoimmune diseases, cardiovascular disease, renal disease, and hypertension and sero-negative for Hepatitis B Virus (HBV) Hepatitis C Virus (HCV), and Human Immunodeficiency Virus (HIV)), non-smokers, non-abusers of drugs or alcohol, non-severely obese (i.e., body mass index (BMI) < 35 kg/m^2^), and not on a restriction diet. All mothers signed an informed consent to participate to the study [25]. The Ethics Committees of participating hospitals approved the study (protocol number: 31,060 MD).

### 2.2. Human Milk Collection and Analysis

Human milk was collected at 6 ± 1 week postpartum. Mothers provided a sample of their milk (30–50 mL) expressed in the morning after breakfast and before lunch. Mothers were instructed to collect their milk at times other than a breastfeeding session, and the times of milk collection as well as of the last breastfeeding were recorded. The freshly expressed foremilk was stirred and divided in sterile 10 mL tubes, overlaid with nitrogen gas to avoid oxidation. Milk samples were then stored at −70 °C.

Human milk macronutrient composition (namely protein, lactose, fat and energy density) was analysed using mid infrared transmission spectroscopy (Human Milk Analyzer, Miris AB, Uppsala, Sweden). The complete profile of FA with chain length between 4 and 24 carbon atoms was analysed as methyl esters by capillary gas chromatography and flame ionization detection (“cGC-FID”) with and without preparative separation of phospholipids by high-performance liquid chromatography and evaporative light-scattering detection (“HPLC-ELSD”) according to the method proposed by Beermann et al. [26].

### 2.3. Maternal Dietary Intake Assessment

Maternal dietary intakes from partum to the day of milk collection were obtained by trained interviewers using a validated and reproducible food frequency questionnaire (FFQ) [27,28], during the same visit in which human milk was collected. The FFQ included information on weekly intake of 78 foods, recipes and non-alcoholic beverages grouped in the following sections: (I) milk and hot beverages; (II) first courses, bread and cereals; (III) second courses (including meat, fish, and cheese); (IV) side dishes (e.g., vegetables); (V) fruits; (VI) sweets, desserts, and carbonated sweet drinks; and (VII) a separate section regarding alcohol frequency and pattern of consumption. Serving size was defined either in “natural” units (e.g., 1 cup of milk, 1 coffee spoonful of sugar, 1 egg, 1 apple) or as small, average, or large according to an Italian average serving size (e.g., 80 g of pasta, 100 g of mixed salad, 175 g of potatoes, 150 g of beef). Other specific items investigated the type of fat (e.g., olive oil, seeds oil, butter) used for cooking or as dressing. Seasonal variations in fruit and vegetable consumption were registered and corresponding average weekly consumptions were re-proportionated. Occasional consumption (i.e., less than once a week and more than once a month) were coded as 0.5 per week. Maternal intakes of macro- and micronutrients, as well as total energy, were estimated using an Italian food composition database [29,30]. In this computation, we weighted the fat composition of each food or recipes according to information on the type of fat used. About half of the participants (52%) in the MEDIDIET study reported using nutritional supplements. However, the only available information was the type of supplement used (i.e., minerals, vitamins, ω-3 fatty acids, probiotics, galactogogues), but not the specific nutritional composition and intake; therefore, we did not include nutritional supplements in the estimate of maternal nutrient intakes. 

### 2.4. Statistical Analyses

**Figure 1:**
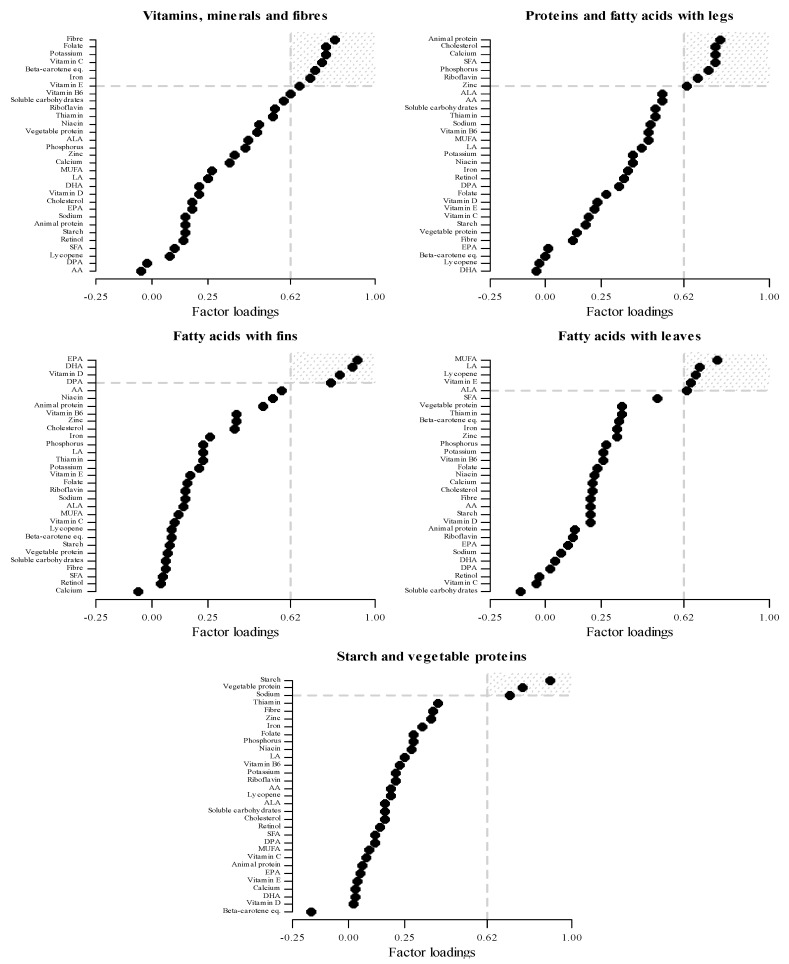
Nutrient’s factor loadings for the five maternal dietary patterns identified in the MEDIDIET study, using principal component factor analysis (PCFA). Estimates from PCFA performed on 31 maternal nutrient intakes. Each nutrient’s factor loading represents how strongly that nutrient is associated with dietary patterns. Nutrient’s factor loadings ≥0.63 represents “dominant nutrients” for each dietary pattern. AA: arachidonic acid; ALA: α-linolenic acid; DHA: docosahexaenoic acid; DPA: docosapentaenoic acid; EPA: eicosapentaenoic acid; LA: linoleic acid; MUFA: monounsaturated fatty acids; SFA: saturated fatty acids.

Maternal dietary patterns were identified through an exploratory principal component factor analysis (PCFA) carried out on the intake of 31 selected nutrients as reported in Figure 1. The nutrients were selected in order to provide an overall representation of diet, including all the caloric components as well as specific FA, minerals and vitamins, and other nutritional compounds. The PCFA is a technique for reducing the complexity and the dimensionality of a set of correlated variables (i.e., nutrients) into a smaller number of uncorrelated and underlying factors (i.e., dietary patterns) in order to enhance interpretability while minimizing information loss [31,32]. We assessed the factorability of the correlation matrix of nutrient intakes by visual inspection, Bartlett’s test of sphericity and individual and overall (Kaiser–Meyer–Olkin) measures of sampling adequacy [33]. The number of dietary patterns was selected considering the scree plot, factor eigenvalues >1, and interpretability. Dietary patterns were orthogonally rotated (Varimax option) to obtain a simpler factor loading structure with greater interpretability. This implies that the obtained dietary patterns are uncorrelated. 

Each nutrient’s factor loading represents how strongly that nutrient is associated with a dietary pattern. We considered as highly associated those nutrients with a factor loading ≥0.63 in absolute value, hereafter named “dominant nutrients”. We set this cut-off because it implies a contribution of the dietary patterns to the original nutrient’s total variance of approximately 40% (i.e., 0.63^2^). The dominant nutrients were used to label the dietary patterns. Mothers’ factor scores were calculated for each dietary pattern as the sum of individual nutrient intakes weighted for the corresponding factor loadings. Each mother’s factor scores indicate to what extent her diet was adherent to each dietary pattern. To further describe and interpret the identified dietary patterns, the Spearman rank correlation coefficients were calculated between the continuous factor scores derived from PCFA and the weekly intake of selected food groups and added fats.

We evaluated the relationship between maternal dietary patterns and foremilk macronutrient and FA components using Pearson’s correlation coefficient. In addition, we evaluated differences in means of human milk compounds across quartiles of maternal dietary patterns (i.e., quartiles on the factor scores) using analysis of variance (ANOVA). We also evaluated the relationship between quartiles of the identified dietary patterns and baseline maternal characteristics (age, BMI, geographical area, tobacco smoking habit). All analyses were performed using SAS software, version 9.4 (SAS Institute, Inc., Cary, NC, USA).

## 3. Results

Table 1 shows the distribution of energy, macronutrient and FA contents in human milk among mothers participating in the MEDIDIET study. 

The energy density ranged from 37.0 to 89.0 kcal/100mL with a mean ± standard deviation (SD) of 57.5 ± 10.5 kcal/100 mL. Regarding macronutrient contents, the lactose content ranged from 6.2 to 7.2 with a mean ± SD of 6.8 ± 0.2 g/100 mL. The corresponding figures were 0.2 (min), 1.4 (max), 0.9 ± 0.2 (mean ± SD) g/100mL for proteins and 0.7 (min), 7.2 (max) and 3.1 ± 1.3 (mean ± SD) g/100 mL for fats. As for the FA contents, the mean ± SD for saturated fatty acids (SFA) was 41.9 ± 4.9% of FA (min = 27.9 and max = 56.1), for monounsaturated fatty acids (MUFA) was 44.1 ± 4.8% of FA (min = 31.1 and max = 60.8) and for polyunsaturated fatty acids (PUFA) was 13.6 ± 2.5% of FA (min = 9.0 and max = 24.3). The mean ± SD contents of ω-6 FA and ω-3 FA were 12.4 ± 2.5 (min = 8.1, max = 23.1)% of FA, and 1.2 ± 0.5 (min = 0.7, max = 4.4)% of FA, respectively.

Visual inspection showed that the correlation matrix of the 31 maternal nutrient intakes was adequate to carry out the PCFA. Supplementary Table S1 shows that the Bartlett’s test of sphericity was statistically significant (*p* < 0.0001), and the overall and individual measures of sampling adequacy were satisfactory (overall measure = 0.86, nine individual measures ≥0.90, 16 individual measures between 0.80 and 0.89, four measures between 0.70 and 0.79, and only two measures <0.70).

Figure 1 and Supplementary Table S2 show nutrient’s factor loadings for the five retained maternal dietary patterns as well as the overall and individual proportion of explained variance by each pattern. Overall, the five dietary patterns explained over 80% of the variance of the maternal nutrient intakes. The first dietary pattern, labelled “Vitamins, minerals, and fibre”, was characterized by high loadings on fibre, folate, potassium, vitamin C, beta-carotene equivalents, iron, and vitamin E. This dietary pattern explained about 22% of the variance of the maternal nutrient intakes. In terms of food groups, this pattern was correlated with a higher maternal consumption of cooked and raw vegetables, non-citrus fruit, olive oil, soups, and low consumption of pasta with meat sauce (Table 2). The “Proteins and fatty acids with legs” dietary pattern had as dominant nutrients animal protein, SFA, cholesterol, calcium, phosphorus, zinc, and riboflavin. This pattern explained 21% of the variance. It was characterized by mothers who had high intakes of milk, cheese, white meat, processed meat, red meat, and butter. The “Fatty acids with fins” dietary pattern had the greatest loadings on eicosapentaenoic acid (EPA), docosahexaenoic acid (DHA), vitamin D, and docosapentaenoic acid (DPA). It explained about 15% of the variance. In terms of foods, this pattern was correlated with maternal consumption of fish, seed oil, white meat, and red meat. The “Fatty acids with leaves” dietary pattern had as dominant nutrients MUFA, linoleic acid (LA), lycopene, vitamin E, and α-linolenic acid (ALA). This pattern explained 12% of the variance. This pattern was characterized by a high maternal consumption of olive oil, plain pasta, raw vegetables, desserts, butter, cooked vegetables, various seed oils, and eggs. The last dietary pattern identified was “Starch and vegetable proteins”, characterized by greatest loadings on starch, vegetable protein, and sodium; it explained 10% of the variance of maternal nutrient intakes and it was correlated with a higher consumption of refined grain bread, plain pasta, pasta with meat, and soups. The communalities of the five selected dietary patterns were generally high—except that of retinol—thus indicating that most original nutrients were fairly represented by the five identified maternal dietary patterns (Supplementary Table S2).

Table 3 shows the correlation coefficients between human milk components and continuous factor scores of the five maternal dietary patterns identified in the MEDIDIET study. No correlations emerged between energy and macronutrients in human milk and the identified dietary patterns, except for a weak positive association between lactose and the “Starch and vegetable proteins” dietary pattern (Pearson’s correlation coefficient r = 0.12). The “Vitamins, minerals and fibre” dietary pattern was positively correlated with the human milk concentrations of ω-3 (r = 0.28), ALA (r = 0.25), EPA (r = 0.23), DPA (r = 0.21) and DHA (r = 0.20); the correlation was negative for AA/EPA ratio (r = −0.27), ω-6/ω-3 ratio (r = −0.23), AA/DHA ratio (r = −0.21), LA/ALA ratio (r = −0.19), and LA/DHA ratio (r = −0.19).

The “Proteins and fatty acids with legs” dietary pattern was weakly positively correlated with human milk content of SFA (r = 0.12) and ω-6 (r = 0.12), and with AA/DHA (r = 0.13) and LA/DHA (r = 0.12) ratios, whereas it was negatively correlated with MUFA (r = −0.19).

There were positive correlations between “Fatty acids with fins” dietary pattern and ω-3 (r = 0.23), EPA (r = 0.25), DHA (r = 0.25), and DPA (r = 0.19), and ALA (r = 0.13) milk contents. The correlation was negative for AA/DHA ratio (r = −0.27), and LA/DHA ratio (r = −0.27), AA/EPA ratio (r = −0.26), ω-6/ω-3 ratio (r = −0.21), and LA/ALA ratio (r = −0.13).

The “Fatty acids with leaves” dietary pattern was positively correlated with MUFA (r = 0.17) and ALA (r = 0.13); it was inversely correlated with SFA (r = −0.20).

Lastly, the “Starch and vegetable proteins” pattern seemed to be positively correlated with lactose (r = 0.12).

Supplementary Table S3 gives the means and SDs of human milk contents across quartiles of the five dietary patterns. These were generally consistent the with aforementioned correlations. In particular, mothers with a high adherence (i.e., 4th quartile) to the “Vitamins, minerals and fibre” dietary pattern showed significantly higher mean contents of ω-3 (*p* = 0.0029), EPA (*p* = 0.0195), DHA (*p* = 0.0093), and DPA (*p* = 0.0273), than mothers with a lower adherence. Conversely, the mean ω-6/ω-3 ratio (*p* = 0.009), AA/EPA ratio (*p* = 0.0012), AA/DHA ratio (*p* = 0.0193), and LA/DHA ratio (*p* = 0.0277) tended to be lower for mothers with a high adherence to this dietary pattern.

An increasing trend of the AA/DHA ratio was observed across quartiles of the “Proteins and fatty acids with legs” dietary pattern (*p* = 0.0378). In addition, although there was no correlation for this component, the mean content of AA was significantly higher in mothers with higher adherence to this dietary pattern (*p* = 0.0473). Although some significant differences emerged for the mean contents of ω-3 (*p* = 0.0339), ALA (*p* = 0.0354), and AA/EPA ratio (*p* = 0.0229) across quartiles of the dietary pattern, the shapes of these relationships were unclear.

Mothers in the highest quartile of the “Fatty acids with fins” dietary pattern showed significantly higher contents of ω-3 (*p* = 0.0038), EPA (*p* = 0.0004), DHA (*p* = 0.0013), and DPA (*p* = 0.0276); conversely, they showed significantly lower ω-6/ω-3 (*p* = 0.0426), AA/EPA (*p* = 0.0004), AA/DHA (*p* = 0.0006), and LA/DHA ratios (*p* = 0.0012).

Mothers in the highest quartiles of the “Fatty acids with leaves” dietary patterns showed significantly higher mean contents of MUFA (*p* = 0.0322) and ALA (*p* = 0.0032), and lower mean content of SFA (*p* = 0.0035) compared to mothers in the lowest quartiles.

No significant difference of the mean content in milk emerged across quartiles of the “Starch and vegetable proteins” dietary pattern.

Supplementary Table S4 shows baseline maternal characteristics and quartiles of factor scores. Some differences emerged according to geographical area. Mothers from Northern and Central Italy had higher adherence to the “Vitamins, minerals and fibre” and the “Fatty acids with leaves” dietary patterns. Mothers form Central and Southern Italy had higher adherence to the “Starch and vegetable proteins” dietary pattern. No other maternal characteristic—including age, BMI, and tobacco smoking habit—appeared to influence the distribution of the identified dietary patterns.

## 4. Discussion

The present study is, to our knowledge, the first Italian investigation considering the role of overall maternal diet on human milk macronutrients and detailed FA composition. In the population under investigation, five dietary patterns were identified based on maternal nutritional intakes during breastfeeding. In particular, the “Vitamins, minerals and fibre” pattern was correlated with higher contents in human milk of ω-3 and its subcomponents (including ALA, EPA, DHA, and DPA). Likewise, higher total ω-3, EPA, DHA, and DPA in milk were correlated with higher adherence to the “Fatty acids with fins” pattern. The “Proteins and fatty acids with legs” pattern was inversely correlated with human milk content of MUFA and weakly positively with SFA, ω-6, AA/DHA ratio, and LA/DHA ratio. Conversely, the “Fatty acids with leaves” dietary pattern was inversely related to the SFA content in human milk, and positively related to the MUFA content in milk. Lastly, the “Starch and vegetable proteins” pattern showed only a weak positive correlation with lactose in milk.

Macronutrient compounds in milk have been reported to vary within mothers and to depend on lactation period, while they appear stable despite variations in maternal nutrition [34]. Conversely, maternal dietary habits play a role in determining human milk lipidic compounds, which include essential fatty acids and provide about 50% of total energy for the infant [35,36]. The most abundant FA in milk are SFA, followed by MUFA and PUFA, and these compounds—expressed as % of FA—are mutually dependent, with an increase in one FA implying a decrease in one or more other FA [35]. Among FA, PUFA tend to be more sensitive to maternal dietary intake [10]. A cross-countries study showed that the proportions of SFA and MUFA were relatively constant across a large number of countries, whereas the level of some PUFA, and particularly DHA, was highly variable, likely due to maternal diet influences [37].

Two previous studies carried out in China have assessed the role of maternal dietary patterns, derived from food groups, on human milk macronutrients [15,20] and FA [15]. To the best of our knowledge no previous study has assessed the role of nutrient-based maternal dietary patterns on human milk compounds. Therefore, comparability between the present and previous studies is limited. In the study of Tian and colleagues, higher proportions of SFA in milk were observed in two dietary patterns characterized by diary, soybean products, and nuts (Pattern 2) and grain/potatoes and beans, and eggs (Pattern 4) [15]. In line with the Pattern 2 of Tian and colleagues, our “Proteins and fatty acids with legs” dietary pattern was characterized by high maternal intakes of milk, cheese and butter, as well as by high intakes of white, red and processed meat, and was correlated with a higher proportion of SFA in human milk. In the same study, higher proportions of PUFA and ω-6 were related to maternal dietary patterns characterized by meats, mushrooms and algae, and marine products (Pattern 1), and vegetables, and fruits (Pattern 3) [15]. In our study, the “Proteins and fatty acids with legs” pattern, also characterized by high intakes of meat, was positively correlated to ω-6 content in milk. In the study of Huang and colleagues, lower protein concentrations in human milk were observed among mothers with higher adherence to a pattern characterized by high intakes of fresh vegetables, fresh legumes, and low intakes of poultry, red meat and eggs; higher adherence to the same pattern was associated with a higher content of lactose in milk [20]. In the same study, adherence to a pattern characterized by high intakes of red meat, cereals, and eggs, and low of milk, nuts and seeds, candy, and fast foods was associated with a higher content of protein in milk. In our study, protein milk content was not related to the identified dietary patterns, and lactose content was only weakly related with a diet high in refined grain bread and pasta, thus no similarities could be found with the study of Huang et al. Besides the difficulty in comparing the studies due to the difference between food-based and nutrient-based dietary patterns, a further complicating factor is the differences in dietary habits between China and Italy.

In a seemingly paradoxical way, we found associations between “Vitamins, minerals and fibre” dietary pattern with concentrations of ω-3 in human milk. Accordingly, the pattern is not characterized by fat-rich components, nor by energy-delivering nutrients. This pattern may be characterized by intakes of the maternal ω-3 (ALA) from vegetable sources, and a generally well balanced food intake within a favorable lifestyle [38]. Within this context, micronutrients may help in improving the PUFA balance of circulating lipids and human milk, as shown by a trial in Cambodian children where a better balance in circulating PUFA levels was associated with a dietary supplementation with a mineral/micronutrient preparation [39]. A cross-countries comparison showed that Italian human milk has among the highest contents of palmitic acid and oleic acid [21]. This is possibly related to the high consumption of olive oil in Italy. In the present study, palmitic acid was inversely correlated with the “Vitamins, minerals and fibre” and the “Fatty acids with leaves” patterns and positively correlated with the “Proteins and fatty acids with legs” pattern. No significant correlation was evident between the oleic acid and the identified dietary patterns (data not shown).

PCFA is a well-known technique used to identify a few a posteriori dietary patterns from a larger set of dietary variables, allowing us to consider diet as an overall exposure, taking into account correlations and interactions between different dietary components. This method requires some subjective decisions in different steps of the analysis, including the type (i.e., foods or nutrients) and number of dietary variables to analyse, the number of factors to retain, the choice of applying a rotation method, and the interpretation of the identified dietary patterns [19]. Thus, we performed some checks and complementary analyses to confirm the choice of the identified dietary patterns and their interpretation. All the nutrients that were included in the PCFA had at least one factor loading >0.30—even those that were not a dominant nutrient on any factor—thus confirming the relevance of all the selected nutrients in the definition of these dietary patterns. Moreover, the standardized Cronbach’s coefficient alphas were calculated for each factor, and coefficient alpha-when-item-deleted (i.e., calculated excluding each nutrient in turn) were calculated for each factor and for each nutrient with factor loading ≥0.40, in order to assess the importance of the nutrients within each dietary pattern. Then, we calculated Spearman rank correlation coefficients between daily intake of selected food groups and continuous factor scores obtained from PCFA, and these supported our interpretation and labelling of the dietary patterns based on nutrients. Moreover, the original dataset was divided in two randomly selected subgroups, PCFA was carried out separately in each subgroup, and this allowed to confirm the dietary patterns identified in the main analysis. In addition, the choice of performing PCFA on nutrients instead of foods would allow the comparability between studies based on different geographical areas, while food-based dietary patterns may be driven by local foods, thus limiting the reproducibility of the results in other settings.

This study has strengths and weaknesses which are inherent to its observational design and general setting. The multicentric approach allowed inclusion of mothers from different Italian regions and living both in inland and coastal areas. Maternal dietary information was self-reported and dependent on participant’s recall. However, it was collected by trained interviewers using a structured FFQ which has been validated and tested for reproducibility in the Italian adult population [27,28], and this allowed us to obtain adequate information on maternal dietary habits during breastfeeding. Moreover, the standardized milk collection and analysis procedures minimized systematic and random errors in the assessment of macronutrients and FA profile in milk. The uniform timing of milk collection (at about six weeks after delivery) allowed us to include all mothers in a mature stage of breastfeeding; furthermore, mothers were recommended to collect their foremilk far away from a breastfeeding session, thus limiting the influence on human milk composition [40]. On the other hand, milk collection in one time point only makes it difficult to speculate on the impact of the maternal diet in the long term. Furthermore, the collection of mature human milk (at about 6 weeks postpartum) may account for the lack of association between maternal diet and the energy and macronutrients in milk.

## 5. Conclusions

In the present study, we identified five major dietary patterns among Italian breastfeeding mothers and assessed their role on human milk macronutrients and FA profile. We confirmed that some human milk compounds—including FA, and in particular ω-3 and its subcomponents—are influenced by maternal dietary habits during breastfeeding. No association was found between maternal dietary patterns and lactose, protein, fat or energy in human milk. Our results suggest that an adequate maternal nutrition during lactation may be important not only for the mother herself, but also to provide the infant with a milk with adequate amounts and quality of nutrients for an appropriate nutrition.

## Figures and Tables

**Table 1:** Distribution of human milk components among 300 breastfeeding mothers. Italy, 2012–2014.

Milk Component	Mean ± SD	Minimum	10th Percentile	Median	90th Percentile	Maximum
Energy (kcal/100mL) ^1^	57.5 ± 10.5	37.0	44.0	56.5	71.5	89.0
Lactose (g/100mL) ^1^	6.8 ± 0.2	6.2	6.6	6.8	7.0	7.2
Protein (g/100mL) ^1^	0.9 ± 0.2	0.2	0.7	0.9	1.1	1.4
Fat (g/100mL) ^1^	3.1 ± 1.3	0.7	1.4	2.9	4.8	7.2
SFA (% of FA) ^2^	41.9 ± 4.9	27.9	36.1	41.9	48.1	56.1
MUFA (% of FA) ^2^	44.1 ± 4.8	31.1	38.0	43.9	50.7	60.8
PUFA (% of FA) ^2^	13.6 ± 2.5	9.0	10.9	13.1	16.9	24.3
ω-6 (% of FA) ^2^	12.4 ± 2.5	8.1	9.8	11.9	15.6	23.1
LA (% of FA) ^2^	10.9 ± 2.4	6.4	8.4	10.4	14.0	21.4
AA (% of FA) ^2^	0.5 ± 0.1	0.3	0.4	0.5	0.6	0.7
ω-3 (% FA) ^2^	1.2 ± 0.5	0.7	0.8	1.1	1.6	4.4
ALA (% of FA) ^2^	0.5 ± 0.2	0.3	0.4	0.5	0.8	1.2
EPA (% of FA) ^2^	0.06 ± 0.05	0.02	0.03	0.04	0.09	0.4
DHA (% of FA) ^2^	0.3 ± 0.2	0.09	0.2	0.2	0.5	2.3
DPA (% of FA) ^2^	0.1 ± 0.06	0.03	0.08	0.1	0.2	0.6
ω-6/ω-3 ratio ^2^	11.2 ± 3.7	2.5	7.2	10.8	16.1	28.3
LA/ALA ratio ^2^	21.9 ± 7.4	5.9	13.6	20.4	31.5	54.1
AA/EPA ratio ^2^	11.2 ± 4.8	1.0	4.7	11.1	17.7	24.6
EPA/DHA ratio ^2^	0.2 ± 0.06	0.08	0.1	0.2	0.3	0.5
AA/DHA ratio ^2^	2.0 ± 0.9	0.2	1.0	1.9	3.2	4.4
LA/DHA ratio ^2^	47.8 ± 24.3	3.7	20.8	45.5	78.9	176.9

^1^ This component was missing in 1 subject. ^2^ This component was missing in 18 subjects. AA: arachidonic acid; ALA: α-linolenic acid; DHA: docosahexaenoic acid; DPA: docosapentaenoic acid; EPA: eicosapentaenoic acid; FA: fatty acids; LA: linoleic acid; MUFA: monounsaturated fatty acids; PUFA: polyunsaturated fatty acids; SD: standard deviation; SFA: saturated fatty acids.

**Table 2:** Spearman rank correlation coefficients between maternal factor scores derived from principal component factor analysis (PCFA) and weekly number of portions of selected food, food groups and seasonings derived on the same data. Italy, 2012–2014.

Food, Food Groups, and Seasoning (Portions/Week)	Vitamins, Minerals and Fibre	Proteins and Fatty Acids with Legs	Fatty Acids with Fins	Fatty Acids with Leaves	Starch and Vegetable Proteins
Milk	0.11	0.50	−0.10	−0.12	0.01
Hot beverages	0.13	−0.12	0.01	0.13	0.12
Added sugars	0.06	0.19	−0.09	−0.21	0.05
Refined grain bread	−0.06	0.01	0.05	−0.11	0.72
Whole grain bread	0.20	−0.06	−0.02	0.15	−0.05
Pizza	−0.10	0.10	0.06	0.05	0.10
Plain pasta	0.09	−0.18	−0.03	0.45	0.30
Pasta with meat	−0.28	0.23	0.20	0.09	0.26
Soup	0.25	0.08	0.12	−0.04	0.25
Eggs	0.07	0.14	−0.01	0.25	0.01
White meat	−0.02	0.28	0.28	−0.05	−0.05
Red meat	0.02	0.27	0.26	0.18	0.08
Pork meat	−0.11	0.10	0.13	0.14	−0.04
Processed meat	−0.20	0.28	0.17	0.15	0.12
Fish	0.18	−0.14	0.77	0.09	−0.04
Cheese	−0.10	0.50	−0.10	0.05	−0.01
Legumes	0.18	0.02	0.00	0.22	0.04
Potatoes	0.13	0.08	0.12	0.08	0.14
Raw vegetables	0.40	−0.02	0.01	0.43	−0.23
Cooked vegetables	0.55	0.03	0.00	0.29	−0.08
Citrus fruit	0.21	0.01	0.07	−0.14	0.13
Non-citrus fruit	0.43	0.07	−0.07	−0.09	0.01
Cooked fruit	0.17	−0.05	0.12	0.00	0.06
Dried fruit	0.18	−0.06	−0.06	0.17	0.10
Cookies	−0.02	0.20	0.00	−0.14	0.17
Desserts	−0.05	0.14	0.07	0.35	0.02
Carbonated sweet drinks	0.01	0.06	0.14	0.21	−0.02
Olive oil	0.35	0.03	0.08	0.67	0.00
Various seeds oils	0.04	0.11	0.29	0.25	0.05
Butter	0.23	0.25	0.03	0.32	−0.03

**Table 3:** Pearson’s correlation coefficients ^1^ between human milk components and continuous factor scores of the five maternal dietary patterns identified in the MEDIDIET study, using principal component factor analysis (PCFA).

	Vitamins, Minerals and Fibres	Proteins and Fatty Acids with Legs	Fatty Acids with Fins	Fatty Acids with Leaves	Starch and Vegetable Proteins
Energy (kcal/100mL) ^2^	−0.09	−0.02	−0.03	0.05	−0.10
Lactose (g/100mL) ^2^	0.00	−0.01	−0.03	0.03	0.12
Protein (g/100mL) ^2^	0.01	0.05	−0.02	0.06	0.09
Fat (g/100mL) ^2^	−0.09	−0.02	−0.03	0.05	−0.10
SFA (% of FA) ^3^	0.00	0.12	−0.06	−0.20	0.01
MUFA (% of FA) ^3^	−0.02	−0.19	0.06	0.17	0.04
PUFA (% of FA) ^3^	0.03	0.11	0.01	0.07	−0.09
ω-6 (% of FA) ^3^	−0.02	0.12	−0.03	0.06	−0.07
LA (% of FA) ^3^	−0.02	0.11	−0.03	0.06	−0.08
AA (% of FA) ^3^	−0.04	0.11	0.06	−0.11	0.02
ω-3 (% FA) ^3^	0.28	−0.05	0.23	0.08	−0.10
ALA (% of FA) ^3^	0.25	−0.01	0.13	0.13	−0.07
EPA (% of FA) ^3^	0.23	−0.06	0.25	0.01	−0.08
DHA (% of FA) ^3^	0.20	−0.10	0.25	0.01	−0.10
DPA (% of FA) ^3^	0.21	−0.01	0.19	0.08	−0.07
ω-6/ω-3 ratio ^3^	−0.23	0.10	−0.21	−0.04	0.05
LA/ALA ratio ^3^	−0.19	0.07	−0.13	−0.08	0.00
AA/EPA ratio ^3^	−0.27	0.04	−0.26	−0.05	0.10
EPA/DHA ratio ^3^	0.11	0.09	0.05	0.05	−0.03
AA/DHA ratio ^3^	−0.21	0.13	−0.27	−0.01	0.10
LA/DHA ratio ^3^	−0.19	0.12	−0.27	0.06	0.03

^1^ Pearson’s correlation coefficient between human milk component and maternal factor score. ^2^ This component was missing in 1 subject. ^3^ This component was missing in 18 subjects. AA: arachidonic acid; ALA: α-linolenic acid; DHA: docosahexaenoic acid; DPA: docosapentaenoic acid; EPA: eicosapentaenoic acid; FA: fatty acids; LA: linoleic acid; MUFA: monounsaturated fatty acids; PUFA: polyunsaturated fatty acids; SD: standard deviation; SFA: saturated fatty acids.

## Data Availability

The data presented in this study are not publicly available. They will be made available upon reasonable request to the corresponding author.

## Supplementary Materials

The following are available online at https://www.mdpi.com/article/10.3390/nu13051722/s1, Table S1: Factorability of the correlation matrix of the maternal nutrient intakes: Bartlett’s test of sphericity and measures of sampling adequacy; Table S2: Nutrient’s factor loadings and explained variance for the five maternal dietary patterns identified in the MEDIDIET study, using principal component factor analysis (PCFA). Table S3: Mean ± SD of human milk contents according to quartiles of the five maternal dietary patterns. Italy, 2012–2014. Table S4: Baseline maternal characteristics and quartiles of the five maternal dietary patterns. Italy, 2012–2014.
