# Supplementary material for: Dietary Patterns of Breastfeeding Mothers and Human Milk Composition: Data from the Italian MEDIDIET Study
Source: Nutrients. 2021 May 19;13(5):1722. doi: 10.3390/nu13051722 (PMC8160768; doi:10.3390/nu13051722)
Supplement: Supplementary file 1 [file nutrients-13-01722-s001.zip › Supplementary Table 1.pdf]

Supplementary Table 1. Factorability of the correlation matrix of the maternal nutrient intakes: Bartlett's test of sphericity and measures of sampling adequacy.

**Bartlett's test of sphericity:** p-value< 0.0001	
**Kaiser-Meyer-Olkin statistic – overall measure of sampling adequacy[1]:** 0.86	
**Individual measures of sampling adequacy:**	
mediocre	lycopene (0.68), retinol (0.58)
middling	soluble carbohydrates (0.78), starch (0.76), DHA (0.71), EPA (0.71)
meritorious	sodium (0.89), vitamin D (0.88), LA (0.88), vegetable protein (0.87), MUFA (0.86), phosphorus (0.86), SFA (0.86), cholesterol (0.86), riboflavin (0.85), calcium (0.85), fibre (0.84), folate (0.84), vitamin E (0.83), vitamin C (0.83), AA (0.80), DPA (0.80)
marvellous	potassium (0.93), vitamin B6 (0.92), iron (0.92), beta-carotene eq. (0.91), niacin (0.90), zinc (0.90), ALA (0.90), thiamin (0.90), animal protein (0.90)

[1]Overall and individual measures of sampling adequacy range between 0 and 1, with values > 0.60 indicating a satisfactory size.

AA: arachidonic acid; ALA: α-linolenic acid; DHA: docosahexaenoic acid; DPA: docosapentaenoic acid; EPA: eicosapentaenoic acid; LA: linoleic acid; MUFA: monounsaturated fatty acids; SFA: saturated fatty acids.
