# Supplementary material for: Dietary Patterns of Breastfeeding Mothers and Human Milk Composition: Data from the Italian MEDIDIET Study
Source: Nutrients. 2021 May 19;13(5):1722. doi: 10.3390/nu13051722 (PMC8160768; doi:10.3390/nu13051722)
Supplement: Supplementary file 1 [file nutrients-13-01722-s001.zip › Supplementary Table 2.pdf]

Supplementary Table 2. Nutrient's factor loadings[1] and explained variance for the five maternal dietary patterns identified in the MEDIDIET study, using principal component factor analysis (PCFA).

Nutrient	Vitamins, minerals and fibres	Proteins and fatty acids with legs	Fatty acids with fins	Fatty acids with leaves	Starch and vegetable proteins	Communalities
Soluble carbohydrates	0.59	0.49	-	-0.11	0.16	0.64
Starch	0.15	0.18	-	0.20	**0.90**	0.92
Animal protein	0.15	**0.78**	0.50	0.13	-	0.90
Vegetable protein	0.47	0.14	-	0.34	**0.78**	0.97
SFA	0.10	**0.76**	-	0.50	0.12	0.85
MUFA	0.27	0.46	0.12	**0.77**	-	0.89
LA	0.25	0.43	0.23	**0.69**	0.25	0.84
AA	-	0.52	0.58	0.20	0.19	0.68
ALA	0.43	0.52	0.14	**0.63**	0.16	0.90
EPA	0.18	-	**0.92**	0.10	-	0.90
DHA	0.21	-	**0.90**	-	-	0.86
DPA	-	0.33	**0.80**	-	0.12	0.76
Cholesterol	0.18	**0.76**	0.37	0.21	0.16	0.81
Fibre	**0.82**	0.12	-	0.20	0.38	0.87
Calcium	0.35	**0.76**	-	0.21	-	0.75
Phoshorus	0.42	**0.73**	0.23	0.27	0.29	0.92
Potassium	**0.78**	0.39	0.21	0.26	0.21	0.92
Iron	**0.71**	0.37	0.26	0.32	0.33	0.92
Zinc	0.37	**0.63**	0.38	0.32	0.37	0.91
Sodium	0.15	0.47	0.15	-	**0.72**	0.79
Retinol	0.14	0.35	-	-	0.14	0.16
Thiamin	0.54	0.49	0.23	0.34	0.40	0.85
Riboflavin	0.55	**0.68**	0.15	0.12	0.21	0.85
Niacin	0.48	0.39	0.54	0.22	0.28	0.79
Vitamin B6	0.62	0.46	0.38	0.26	0.23	0.87
Folate	**0.78**	0.27	0.16	0.23	0.29	0.85
Vitamin C	**0.76**	0.19	0.10	-	-	0.64
Vitamin D	0.21	0.23	**0.84**	0.20	-	0.84
Vitamin E	**0.66**	0.22	0.17	**0.65**	-	0.93
Beta-carotene eq.	**0.73**	-	-	0.33	-0.17	0.68
Lycopene	-	-	-	**0.67**	0.19	0.87
**Explained variance (%)**	**21.77**	**21.05**	**15.38**	**12.07**	**10.31**	
**Cumulative explained variance (%)**	**21.77**	**42.82**	**58.19**	**70.26**	**80.57**	

[1]Estimates from a PCFA performed on 31 maternal nutrient intakes. Each nutrient's factor loading represents how strongly that nutrient is associated with dietary patterns. Nutrient's factor loadings ≥0.63 represented "dominant nutrients" for each dietary pattern, and were shown in bold typeface; nutrient's factor loadings smaller than 0.1 were suppressed.

AA: arachidonic acid; ALA: α-linolenic acid; DHA: docosahexaenoic acid; DPA: docosapentaenoic acid; EPA: eicosapentaenoic acid; LA: linoleic acid.
