# Supplementary material for: Dietary Patterns of Breastfeeding Mothers and Human Milk Composition: Data from the Italian MEDIDIET Study
Source: Nutrients. 2021 May 19;13(5):1722. doi: 10.3390/nu13051722 (PMC8160768; doi:10.3390/nu13051722)
Supplement: Supplementary file 1 [file nutrients-13-01722-s001.zip › Supplementary Table 3.pdf]

Supplementary Table 3. Mean ± SD of human milk contents according to quartiles of the five maternal dietary patterns. Italy, 2012-2014.

Milk component	Q1	Q2	Q3	Q4	p-value[1]
	**Vitamins, minerals and fibre**				
	mean (SD)	mean (SD)	mean (SD)	mean (SD)	
Energy (kcal/100ml)[2]	58.21 (12.14)	58.74 (8.94)	56.33 (9.63)	56.67 (10.85)	0.4253
Lactose (g/100ml)[2]	6.79 (0.17)	6.8 (0.13)	6.82 (0.15)	6.80 (0.18)	0.8373
Protein (g/100ml)[2]	0.87 (0.19)	0.9 (0.17)	0.94 (0.16)	0.88 (0.19)	0.1012
Fat (g/100ml)[2]	3.18 (1.52)	3.22 (1.10)	2.92 (1.19)	2.99 (1.38)	0.4293
SFA (% of FA)[3]	41.87 (4.38)	42.63 (4.58)	41.92 (5.03)	41.22 (5.60)	0.4132
MUFA (% of FA)[3]	44.00 (4.41)	43.47 (4.49)	44.14 (4.95)	44.63 (5.36)	0.5631
PUFA (% of FA)[3]	13.66 (2.39)	13.48 (2.56)	13.52 (2.31)	13.72 (2.87)	0.9352
ω-6 (% of FA)[3]	12.54 (2.39)	12.37 (2.53)	12.22 (2.31)	12.40 (2.83)	0.9001
LA (% of FA)[3]	11.03 (2.34)	10.91 (2.40)	10.74 (2.29)	10.88 (2.75)	0.9140
AA (% of FA)[3]	0.48 (0.09)	0.46 (0.08)	0.46 (0.08)	0.48 (0.08)	0.1229
ω-3 (% FA)[3]	1.12 (0.24)	1.11 (0.35)	1.29 (0.63)	1.32 (0.44)	0.0029
ALA (% of FA)[3]	0.52 (0.15)	0.51 (0.16)	0.54 (0.17)	0.58 (0.17)	0.0507
EPA (% of FA)[3]	0.05 (0.02)	0.05 (0.04)	0.06 (0.06)	0.07 (0.05)	0.0195
DHA (% of FA)[3]	0.25 (0.1)	0.25 (0.13)	0.35 (0.36)	0.32 (0.18)	0.0093
DPA (% of FA)[3]	0.11 (0.03)	0.11 (0.04)	0.13 (0.08)	0.13 (0.05)	0.0273
ω-6/ω-3 ratio[3]	11.76 (3.32)	11.97 (3.59)	10.75 (4.19)	10.12 (3.39)	0.009
LA/ALA ratio[3]	22.61 (6.51)	23.08 (7.65)	21.56 (7.59)	20.05 (7.61)	0.0780
AA/EPA ratio[3]	12.41 (4.24)	12.13 (5.32)	10.38 (4.69)	9.71 (4.44)	0.0012
EPA/DHA ratio[3]	0.18 (0.06)	0.20 (0.08)	0.19 (0.06)	0.20 (0.07)	0.2321
AA/DHA ratio[3]	2.17 (0.80)	2.15 (0.84)	1.86 (0.93)	1.83 (0.77)	0.0193
LA/DHA ratio[3]	50.86 (23.28)	52.35 (23.22)	46.09 (30.12)	41.12 (17.22)	0.0277
	**Proteins and fatty acids with legs**				
Energy (kcal/100ml)[2]	57.15 (10.19)	58.44 (10.98)	56.41 (9.23)	57.94 (11.36)	0.6533
Lactose (g/100ml)[2]	6.81 (0.14)	6.80 (0.15)	6.79 (0.19)	6.81 (0.16)	0.9086
Protein (g/100ml)[2]	0.88 (0.19)	0.88 (0.17)	0.92 (0.17)	0.90 (0.19)	0.5100
Fat (g/100ml)[2]	3.04 (1.27)	3.18 (1.35)	2.95 (1.16)	3.13 (1.43)	0.7157
SFA (% of FA)[3]	41.19 (5.25)	41.91 (5.57)	42.28 (3.56)	42.30 (5.05)	0.5098
MUFA (% of FA)[3]	45.00 (5.30)	44.23 (5.11)	43.67 (4.00)	43.28 (4.61)	0.1709
PUFA (% of FA)[3]	13.41 (2.50)	13.42 (2.38)	13.60 (2.46)	13.95 (2.77)	0.5594
ω-6 (% of FA)[3]	12.12 (2.40)	12.24 (2.37)	12.50 (2.45)	12.69 (2.81)	0.5422
LA (% of FA)[3]	10.66 (2.34)	10.77 (2.30)	10.97 (2.40)	11.17 (2.71)	0.6330
AA (% of FA)[3]	0.45 (0.08)	0.46 (0.08)	0.48 (0.09)	0.49 (0.09)	0.0473
ω-3 (% FA)[3]	1.29 (0.60)	1.18 (0.31)	1.09 (0.25)	1.26 (0.52)	0.0339
ALA (% of FA)[3]	0.56 (0.18)	0.52 (0.14)	0.50 (0.14)	0.57 (0.18)	0.0354
EPA (% of FA)[3]	0.07 (0.06)	0.05 (0.03)	0.05 (0.03)	0.06 (0.04)	0.0559
DHA (% of FA)[3]	0.34 (0.29)	0.30 (0.14)	0.24 (0.10)	0.30 (0.28)	0.0640
DPA (% of FA)[3]	0.13 (0.07)	0.11 (0.04)	0.11 (0.04)	0.13 (0.07)	0.3864
ω-6/ω-3 ratio[3]	10.59 (3.76)	10.97 (3.29)	12.01 (3.88)	11.09 (3.77)	0.1280
LA/ALA ratio[3]	20.93 (8.01)	21.97 (7.08)	23.32 (7.37)	21.14 (7.01)	0.2058
AA/EPA ratio[3]	10.48 (5.36)	10.47 (4.15)	12.61 (4.87)	11.16 (4.56)	0.0229
EPA/DHA ratio[3]	0.20 (0.08)	0.18 (0.05)	0.19 (0.06)	0.20 (0.06)	0.3407
AA/DHA ratio[3]	1.86 (0.92)	1.87 (0.85)	2.17 (0.68)	2.14 (0.91)	0.0378
LA/DHA ratio[3]	44.18 (24.59)	44.57 (21.99)	51.89 (24.92)	50.35 (25.03)	0.1303
	**Fatty acids with fins**				

Energy (kcal/100ml)[2]	58.71 (9.69)	56.49 (10.47)	57.05 (10.53)	57.68 (11.15)	0.6041
Lactose (g/100ml)[2]	6.81 (0.14)	6.81 (0.17)	6.79 (0.18)	6.80 (0.16)	0.8654
Protein (g/100ml)[2]	0.91 (0.19)	0.88 (0.15)	0.93 (0.19)	0.87 (0.18)	0.1766
Fat (g/100ml)[2]	3.21 (1.20)	2.96 (1.30)	3.03 (1.31)	3.11 (1.40)	0.6777
SFA (% of FA)[3]	42.21 (4.34)	42.24 (4.91)	42.07 (4.91)	41.25 (5.36)	0.5767
MUFA (% of FA)[3]	43.68 (4.38)	44.09 (4.72)	43.75 (4.64)	44.60 (5.37)	0.6445
PUFA (% of FA)[3]	13.65 (2.56)	13.23 (2.42)	13.76 (2.53)	13.72 (2.60)	0.5797
ω-6 (% of FA)[3]	12.53 (2.61)	12.11 (2.38)	12.53 (2.48)	12.37 (2.58)	0.7307
LA (% of FA)[3]	11.04 (2.50)	10.60 (2.32)	11.03 (2.41)	10.89 (2.52)	0.6942
AA (% of FA)[3]	0.46 (0.09)	0.47 (0.08)	0.47 (0.09)	0.48 (0.08)	0.6545
ω-3 (% FA)[3]	1.12 (0.36)	1.11 (0.27)	1.23 (0.43)	1.35 (0.60)	0.0038
ALA (% of FA)[3]	0.53 (0.17)	0.51 (0.14)	0.53 (0.17)	0.57 (0.18)	0.1014
EPA (% of FA)[3]	0.04 (0.03)	0.05 (0.02)	0.06 (0.05)	0.07 (0.06)	0.0004
DHA (% of FA)[3]	0.23 (0.12)	0.25 (0.12)	0.31 (0.20)	0.36 (0.33)	0.0013
DPA (% of FA)[3]	0.11 (0.05)	0.11 (0.03)	0.12 (0.05)	0.14 (0.08)	0.0276
ω-6/ω-3 ratio[3]	12.05 (4.09)	11.42 (3.33)	11.06 (3.47)	10.31 (3.75)	0.0426
LA/ALA ratio[3]	22.57 (7.77)	22.11 (6.58)	22.35 (7.76)	20.56 (7.41)	0.3474
AA/EPA ratio[3]	12.62 (4.66)	12.06 (4.46)	10.84 (4.85)	9.50 (4.72)	0.0004
EPA/DHA ratio[3]	0.19 (0.06)	0.19 (0.06)	0.19 (0.07)	0.20 (0.07)	0.4675
AA/DHA ratio[3]	2.29 (0.87)	2.15 (0.75)	1.87 (0.85)	1.77 (0.84)	0.0006
LA/DHA ratio[3]	56.67 (28.11)	49.22 (21.68)	45.43 (23.66)	40.98 (21.26)	0.0012
	**Fatty acids with leaves**				
Energy (kcal/100ml)[2]	56.92 (11.09)	57.97 (9.1)	56.56 (10.61)	58.50 (10.96)	0.6446
Lactose (g/100ml)[2]	6.79 (0.16)	6.80 (0.16)	6.81 (0.19)	6.81 (0.13)	0.8652
Protein (g/100ml)[2]	0.88 (0.17)	0.90 (0.20)	0.88 (0.17)	0.92 (0.18)	0.6361
Fat (g/100ml)[2]	3.02 (1.40)	3.13 (1.13)	2.96 (1.32)	3.19 (1.36)	0.7139
SFA (% of FA)[3]	43.29 (5.1)	42.54 (4.69)	41.30 (5.28)	40.54 (4.06)	0.0035
MUFA (% of FA)[3]	43.14 (4.99)	43.36 (4.52)	44.49 (4.70)	45.21 (4.75)	0.0322
PUFA (% of FA)[3]	13.11 (2.57)	13.67 (2.46)	13.79 (2.51)	13.80 (2.54)	0.3194
ω-6 (% of FA)[3]	12.03 (2.52)	12.44 (2.45)	12.53 (2.54)	12.54 (2.53)	0.5703
LA (% of FA)[3]	10.52 (2.47)	10.93 (2.39)	11.02 (2.43)	11.10 (2.46)	0.5012
AA (% of FA)[3]	0.47 (0.08)	0.48 (0.09)	0.48 (0.09)	0.45 (0.07)	0.0637
ω-3 (% FA)[3]	1.09 (0.23)	1.23 (0.53)	1.25 (0.47)	1.26 (0.47)	0.0735
ALA (% of FA)[3]	0.49 (0.14)	0.52 (0.16)	0.57 (0.18)	0.58 (0.17)	0.0032
EPA (% of FA)[3]	0.05 (0.02)	0.06 (0.06)	0.06 (0.05)	0.05 (0.04)	0.1971
DHA (% of FA)[3]	0.26 (0.10)	0.32 (0.26)	0.30 (0.20)	0.29 (0.28)	0.3732
DPA (% of FA)[3]	0.11 (0.03)	0.13 (0.06)	0.12 (0.06)	0.12 (0.07)	0.1001
ω-6/ω-3 ratio[3]	11.49 (3.06)	11.35 (4.42)	11.03 (3.82)	10.83 (3.39)	0.7085
LA/ALA ratio[3]	22.79 (7.01)	23.08 (8.77)	21.00 (6.79)	20.57 (6.64)	0.1068
AA/EPA ratio[3]	11.70 (4.49)	10.93 (5.23)	11.22 (5.06)	10.94 (4.48)	0.7588
EPA/DHA ratio[3]	0.19 (0.07)	0.19 (0.06)	0.20 (0.07)	0.19 (0.06)	0.7609
AA/DHA ratio[3]	2.05 (0.72)	1.95 (0.91)	2.08 (0.96)	1.96 (0.80)	0.7777
LA/DHA ratio[3]	45.91 (17.85)	46.08 (27.57)	49.80 (28.13)	49.32 (22.34)	0.6746
	**Starch and vegetable proteins**				
Energy (kcal/100ml)[2]	57.92 (9.93)	57.80 (10.85)	58.62 (11.13)	55.61 (9.81)	0.3238
Lactose (g/100ml)[2]	6.79 (0.16)	6.79 (0.15)	6.80 (0.17)	6.83 (0.16)	0.3826
Protein (g/100ml)[2]	0.91 (0.21)	0.88 (0.18)	0.87 (0.17)	0.93 (0.15)	0.2088
Fat (g/100ml)[2]	3.13 (1.22)	3.11 (1.35)	3.22 (1.41)	2.84 (1.22)	0.3011
SFA (% of FA)[3]	42.30 (6.03)	41.29 (4.64)	41.66 (4.12)	42.47 (4.69)	0.4538
MUFA (% of FA)[3]	43.71 (5.58)	44.30 (4.82)	44.17 (4.38)	43.99 (4.42)	0.9003

PUFA (% of FA)[3]	13.53 (2.34)	13.99 (2.77)	13.74 (2.70)	13.10 (2.19)	0.2011
ω-6 (% of FA)[3]	12.24 (2.32)	12.76 (2.73)	12.58 (2.66)	11.96 (2.26)	0.2378
LA (% of FA)[3]	10.77 (2.30)	11.25 (2.65)	11.09 (2.58)	10.44 (2.13)	0.2052
AA (% of FA)[3]	0.46 (0.09)	0.47 (0.07)	0.47 (0.08)	0.47 (0.10)	0.9371
ω-3 (% FA)[3]	1.29 (0.58)	1.23 (0.39)	1.16 (0.40)	1.14 (0.38)	0.1966
ALA (% of FA)[3]	0.55 (0.18)	0.54 (0.17)	0.53 (0.16)	0.52 (0.15)	0.7468
EPA (% of FA)[3]	0.06 (0.05)	0.06 (0.04)	0.05 (0.05)	0.05 (0.03)	0.3639
DHA (% of FA)[3]	0.34 (0.34)	0.31 (0.18)	0.27 (0.16)	0.27 (0.14)	0.1378
DPA (% of FA)[3]	0.13 (0.08)	0.12 (0.04)	0.12 (0.05)	0.11 (0.05)	0.4400
ω-6/ω-3 ratio[3]	10.55 (3.90)	11.23 (3.82)	11.55 (3.38)	11.33 (3.71)	0.4126
LA/ALA ratio[3]	21.19 (7.52)	22.39 (7.86)	22.35 (7.52)	21.51 (6.75)	0.7053
AA/EPA ratio[3]	10.38 (4.66)	10.65 (4.44)	11.47 (4.85)	12.25 (5.13)	0.0910
EPA/DHA ratio[3]	0.19 (0.07)	0.19 (0.06)	0.20 (0.07)	0.18 (0.05)	0.6127
AA/DHA ratio[3]	1.83 (0.78)	1.96 (0.87)	2.09 (0.78)	2.15 (0.94)	0.1282
LA/DHA ratio[3]	44.50 (26.08)	48.11 (26.07)	48.98 (18.36)	49.37 (26.19)	0.6272

[1]p-value from ANOVA comparing means across quartiles of the dietary pattern. [2]This component was missing in 1 subject. [3]This component was missing in 18 subjects.

AA: arachidonic acid; ALA: α-linolenic acid; DHA: docosahexaenoic acid; DPA: docosapentaenoic acid; EPA: eicosapentaenoic acid; FA: fatty acids; LA: linoleic acid; MUFA: monounsaturated fatty acids; PUFA: polyunsaturated fatty acids; Q1: 1st quartile of the dietary pattern; Q2: 2nd quartile of the dietary pattern; Q3: 3rd quartile of the dietary pattern; Q4: 4th quartile of the dietary pattern; SD: standard deviation; SFA: saturated fatty acids.
