# Supplementary material for: Dietary Patterns of Breastfeeding Mothers and Human Milk Composition: Data from the Italian MEDIDIET Study
Source: Nutrients. 2021 May 19;13(5):1722. doi: 10.3390/nu13051722 (PMC8160768; doi:10.3390/nu13051722)
Supplement: Supplementary file 1 [file nutrients-13-01722-s001.zip › Supplementary Table 4.pdf]

Supplementary table 4. Baseline maternal characteristics and quartiles of the five maternal dietary patterns. Italy, 2012-2014.

	Age	BMI	Geographical area			Tobacco smoking[1]	
	mean (SD)	mean (SD)	North, %	Center, %	South, %	Never smokers, %	Former smokers, %
			Tor	Fir Rom	SGR Pal		
**Vitamins, minerals and fibre**							
I quartile	32.0 (3.7)	22.8 (3.7)	24.55	20.29	28.10	27.47	21.19
II quartile	32.5 (4.1)	22.3 (3.2)	22.73	15.94	32.23	23.63	27.12
III quartile	32.9 (4.1)	22.2 (2.9)	25.45	23.19	25.62	23.63	27.12
IV quartile	33.1 (4.4)	21.9 (3.0)	27.27	40.58	14.05	25.27	24.58
p-value[2]	*0.3417*	*0.3241*		*0.0042*		*0.0654*	
**Proteins and fatty acids with legs**							
I quartile	32.9 (4.0)	22.3 (3.2)	28.18	24.64	22.31	24.73	25.42
II quartile	32.8 (4.3)	22.6 (3.3)	25.45	23.19	25.62	26.37	22.88
III quartile	32.1 (4.0)	21.9 (2.9)	19.09	28.99	28.10	24.18	26.27
IV quartile	32.6 (4.2)	22.5 (3.4)	27.27	23.19	23.97	24.73	25.42
p-value[2]	*0.5747*	*0.5355*		*0.7137*		*0.9183*	
**Fatty acids with fins**							
I quartile	32.0 (3.7)	22.0 (3.2)	28.18	23.19	23.14	25.82	23.73
II quartile	32.6 (4.3)	22.3 (3.2)	28.18	17.39	26.45	26.92	22.03
III quartile	32.7 (4.4)	22.5 (3.4)	21.82	23.19	28.93	23.63	27.12
IV quartile	33.1 (4.1)	22.4 (3.2)	21.82	36.23	21.49	23.63	27.12
p-value[2]	*0.3417*	*0.8181*		*0.1940*		*0.6803*	
**Fatty acids with leaves**							
I quartile	32.0 (4.1)	22.5 (3.0)	13.64	18.84	38.84	26.37	22.88
II quartile	32.0 (3.9)	22.3 (3.6)	18.18	20.29	33.88	26.92	22.03
III quartile	33.3 (4.4)	22.8 (3.1)	33.64	24.64	17.36	23.08	27.97
IV quartile	33.2 (3.9)	21.7 (3.0)	34.55	36.23	9.92	23.63	27.12
p-value[2]	*0.0734*	*0.1893*		*<0.0001*		*0.5585*	
**Starch and vegetable proteins**							
I quartile	32.6 (4.4)	22.3 (2.9)	32.73	20.29	20.66	27.47	21.19
II quartile	32.6 (3.3)	21.8 (3.1)	29.09	24.64	21.49	24.18	26.27
III quartile	32.8 (4.2)	22.8 (3.5)	23.64	24.64	26.45	24.18	26.27
IV quartile	32.5 (4.5)	22.3 (3.3)	14.55	30.43	31.40	24.18	26.27
p-value[2]	*0.9656*	*0.3244*		*0.0387*		*0.6803*	

[1] Being current smokers was an exclusion criterion, participants were categorized in never vs former smokers. [2]p-value for age and BMI were from ANOVA; p-value for geographical area and tobacco smoking were from chi-square test.

BMI: body mass index, SD: standard deviation
